# Obesity and Endothelial Function

**DOI:** 10.3390/biomedicines10071745

**Published:** 2022-07-19

**Authors:** Masato Kajikawa, Yukihito Higashi

**Affiliations:** 1 Division of Regeneration and Medicine, Medical Center for Translational and Clinical Research, Hiroshima University Hospital, 1-2-3 Kasumi, Minami-ku, Hiroshima 734-8551, Japan; m-kajikawa@hiroshima-u.ac.jp; 2 Department of Regenerative Medicine, Division of Radiation Medical Science, Research Institute for Radiation Biology and Medicine, Hiroshima University, 1-2-3 Kasumi, Minami-ku, Hiroshima 734-8551, Japan

**Keywords:** obesity, endothelial function, atherosclerosis, cardiovascular events

## Abstract

Obesity is a major public health problem and is related to increasing rates of cardiovascular morbidity and mortality. Over 1.9 billion adults are overweight or obese worldwide and the prevalence of obesity is increasing. Obesity influences endothelial function through obesity-related complications such as hypertension, dyslipidemia, diabetes, metabolic syndrome, and obstructive sleep apnea syndrome. The excess fat accumulation in obesity causes adipocyte dysfunction and induces oxidative stress, insulin resistance, and inflammation leading to endothelial dysfunction. Several anthropometric indices and imaging modalities that are used to evaluate obesity have demonstrated an association between obesity and endothelial function. In the past few decades, there has been great focus on the mechanisms underlying endothelial dysfunction caused by obesity for the prevention and treatment of cardiovascular events. This review focuses on pathophysiological mechanisms of obesity-induced endothelial dysfunction and therapeutic targets of obesity.

## 1. Introduction

Obesity is one of the major public health problems due to its effects on morbidity, mortality, and healthcare costs. Body mass index (BMI) is a universally recognized parameter for classifying obesity. The World Health Organization defines overweight as a BMI of 25.0 to 29.9 kg/m^2^ and obesity as a BMI of ≥30.0 kg/m^2^ [1]. The prevalence of obesity has been increasing worldwide over the past few decades [2]. Over 1.9 billion adults worldwide were overweight, and more than 650 million adults were obese in 2016 [3]. Obesity increases the risk of hypertension, dyslipidemia, type 2 diabetes, and cardiovascular events [4,5]. The Global Burden of Disease investigators reported that a high BMI contributed to 4.0 million deaths (2.7 million cardiovascular deaths) in 2015 [2]. Obesity has attracted considerable attention as a worldwide public health problem.

Endothelial dysfunction is the initial step in the pathogenesis and development of atherosclerosis [6,7]. There is solid evidence that obesity induces endothelial dysfunction [3]. Therefore, great attention has been paid to the mechanisms underlying endothelial dysfunction caused by obesity for the prevention and treatment of cardiovascular events. This review focuses on the pathophysiological mechanisms of obesity-induced endothelial dysfunction and potential therapeutic targets of obesity for the prevention of endothelial dysfunction.

## 2. Endothelial Function

**Figure 1:**
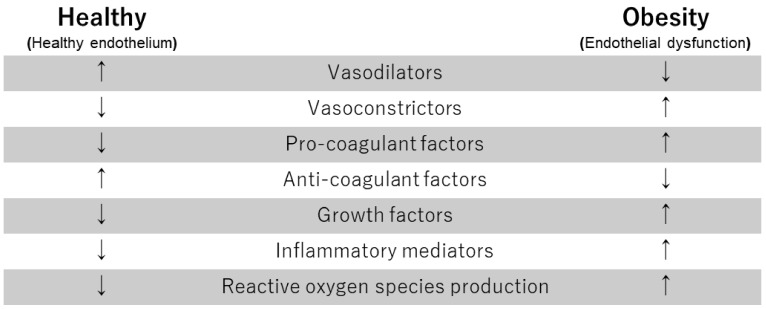
The function of endothelial cells in healthy subjects and obese subjects.

The vascular endothelium is an interface between circulating blood and blood vessels. The vascular endothelium senses shear stress generated by blood flow and secretes vasodilating substances (e.g., nitric oxide (NO), prostacyclin, and endothelium-derived hyperpolarizing factor) and vasoconstricting substances (e.g., endothelin-1, angiotensin II and thromboxane A2) as a paracrine organ [8,9]. Shear stress is essential for regulating cellular signaling in the endothelium. Among the vasoactive agents, NO plays a critical role in maintaining vascular homeostasis, such as endothelial function, blood pressure, and blood flow. The vascular endothelium maintains vascular homeostasis through the regulation of the balances between vasoconstriction and vasodilation, growth promotion and growth inhibition, pro-thrombosis and anti-thrombosis, pro-inflammation and anti-inflammation, and pro-oxidation and anti-oxidation (Figure 1) [8,9]. Endothelial dysfunction has been characterized by enhanced endothelium-dependent contraction and decreased endothelium-dependent relaxation [10]. Endothelial dysfunction is an early stage in the development of atherosclerosis leading to an increased risk of cardiovascular events [6,7]. It is well known that endothelial function is impaired in patients with coronary risk factors. Accumulating evidence indicates that endothelial dysfunction is an independent predictor of cardiovascular events [11,12,13]. Increased NO inactivation and/or reduced NO production by reactive oxygen species (ROS), inflammation, an imbalance between vasodilators and vasoconstrictors, endogenous endothelial NO synthase (eNOS) uncoupling, and low shear stress are important mechanisms that lead to endothelial dysfunction [7].

## 3. Association of BMI with Endothelial Function in Clinical Settings

**Figure 2:**
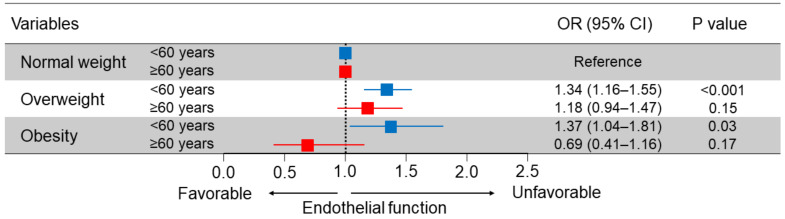
Odds ratios and 95% confidence intervals, adjusted for the presence of hypertension, dyslipidemia, diabetes, and smokers, for endothelial dysfunction according to obesity status. The subjects with a lower quartile of flow-mediated vasodilation (FMD) were defined as subjects having endothelial dysfunction (FMD of less than 4.2% in subjects aged <60 years, FMD of less than 2.1% in subjects aged ≥60 years). Reprinted with permission from Ref. [5]. Copyright 2021 Elsevier.

A significant relationship between BMI and endothelial function has been shown in a clinical setting. A high BMI has been shown to be associated with endothelial dysfunction in children [14], adolescents [15], healthy subjects [16], the general population [5], patients with suspected coronary artery disease [17], and patients with cardiovascular disease [18]. A large cross-sectional study (*n* = 7682) was conducted for evaluating the association of high BMI with endothelial function [5]. The study revealed that BMI can be considered a reliable predictive factor of endothelial dysfunction. Indeed, a positive correlation has been shown between the risk of endothelial dysfunction and obesity and overweight conditions in younger adults (<60 years of age) (Figure 2). However, in older adults (≥60 years of age) no significant differences were found between flow-mediated vasodilation (FMD) values in the three different groups (obese, overweight, and normal weight) (Figure 2) [5]. A prospective longitudinal study demonstrated that a high BMI in childhood and trajectories of BMI from childhood to early midlife are associated with endothelial dysfunction in early midlife [19]. The contribution of obesity to endothelial dysfunction was greater in younger adults than in older adults and a high BMI in childhood induced sustained endothelial dysfunction [19]. In addition, a previous large cohort study (*n* = 324135) has shown that the relative risk of mortality from cardiovascular disease associated with excess weight is higher in younger adults than in older adults [20]. These findings suggest that the obesity paradox in older adults exists in both endothelial function and cardiovascular mortality.

## 4. Visceral Adiposity and Endothelial Function

**Figure 3:**
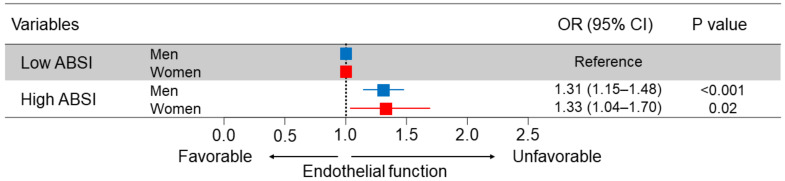
Odds ratios and 95% confidence intervals, adjusted for age, body mass index, presence of hypertension, dyslipidemia, and diabetes, and smokers, for endothelial dysfunction of high ABSI. The subjects with endothelial dysfunction were defined as follows: Men, FMD of less than 3.6%, Women, FMD of less than 3.1%. Low ABSI was defined as follows: Men, less than 0.0796, Women, less than 0.0823. Adapted from [28] 2021 Springer Nature Limited.

Calculation of BMI is a simple method for the assessment of obesity. However, BMI does not accurately predict body fat distribution. It has been shown that abdominal obesity is more strongly associated than overall obesity with cardiovascular events [21,22]. Therefore, the assessment of abdominal obesity in conjunction with BMI is recommended to evaluate cardiovascular risk [23,24]. Indeed, a number of studies have focused on the associations of indices of abdominal obesity with endothelial function. Endothelial function has been shown to be associated with waist circumference [25], waist-to-hip ratio [26], waist-to-height ratio [27], body adiposity index [25], and a body shape index (ABSI) [28]. Recently, we have reported that ABSI is negatively correlated with FMD and high ABSI is an independent predictor of endothelial dysfunction. These findings suggest that ABSI is a useful tool for evaluating cardiovascular risk (Figure 3) [28].

Advances in imaging techniques such as computed tomography (CT) and magnetic resonance imaging have led to in-depth knowledge of the association of body fat distribution with cardiovascular risk. It has been demonstrated that the regional distribution of body fat has a greater influence than fat mass per se on cardiometabolic risk [29,30]. There are two main abdominal adipose compartments: visceral adipose tissue and subcutaneous adipose tissue. Accumulating data have suggested that visceral adiposity is associated with chronic inflammation, insulin resistance, and cardiovascular disease, while subcutaneous adiposity may be protective in this context [30,31]. An increased volume of visceral adipose tissue has been shown to be associated with endothelial dysfunction [32,33,34]. On the other hand, it was shown that the volume of subcutaneous adipose tissue was not associated with endothelial function [33,34]. Romero-Corral et al. showed that visceral fat gain was negatively correlated with FMD (rho = −0.42, *p* = 0.004) but not subcutaneous fat gain (rho = −0.22, *p* = 0.15) in normal-weight healthy young subjects who gained approximately 4 kg [35]. These findings suggest that visceral fat accumulation is a stronger risk factor than subcutaneous fat accumulation for endothelial dysfunction.

Excess visceral adiposity is frequently accompanied by ectopic fat accumulation such as fat accumulation in the liver, heart, and blood vessels [36]. It has been demonstrated that endothelial function is impaired in patients with non-alcoholic fatty liver disease [37], patients with increased epicardial adipose tissue [38,39], and subjects with increased perivascular adipose tissue [40]. It has been suggested that ectopic fat deposition is associated with insulin resistance and an increased risk of cardiovascular complications [41,42]. The major organ of ectopic fat accumulation in the liver and fatty liver is thought to have a critical role in the pathogenesis of cardiovascular disease complications [43]. However, it remains unknown whether fatty liver, per se, is independently associated with cardiovascular events. *PNPLA3 1148M* and *TM6SF2 E167K* variants are genetic factors for the development of fatty liver [44,45]. A recent Mendelian randomization study has shown that these genetically high liver fat contents are not associated with a risk of ischemic heart disease [41]. Further studies are needed to evaluate the pathophysiological significance of fatty liver for endothelial dysfunction. 

The blood vessels, heart, and coronary arteries are surrounded by adipose tissue. Although adipose tissue is considered to be the supporting tissue for perivascular and epicardial regions, its importance has attracted scientific attention because of its anatomical location. It has been recognized that perivascular and epicardial adipose tissue depots have protective effects on endothelial function under physiological conditions by secretion of anti-inflammatory adipokines [46,47]. In obesity, the excessive accumulation of perivascular and epicardial adipose tissues causes adipose tissue dysfunction [48]. Dysfunctional adipose tissue increases the production of pro-inflammatory adipokines and cytokines, leading to endothelial dysfunction [48]. Adipose tissue transplant studies in mice demonstrated that perivascular adipose tissue harvested from obese mice promotes inflammation and endothelial dysfunction in recipient mice [49,50]. In addition, a number of clinical studies have suggested links between ectopic fat deposition and endothelial function [38,39,40,42,51,52,53]. The thickness of epicardial fat has been shown to be associated with endothelial dysfunction [38,52]. In clinical studies, perivascular and epicardial adipose tissues from obese subjects who underwent cardiothoracic surgery showed upregulated production of pro-inflammatory cytokines [53,54]. These findings suggest that perivascular and epicardial adipose tissues are an important source of inflammation and contribute to the pathogenesis of endothelial dysfunction in obesity.

## 5. Adipose Tissue

Adipose tissue is distributed throughout the body and plays an important role in fuel storage and the maintenance of energy balance. Adipose tissue secretes adiponectin, cytokines, and chemokines that are involved in metabolism and inflammation at local and systemic levels [55]. In mammals, there are two major types of adipose tissue, categorized as white adipose tissue and brown adipose tissue. White adipose tissue acts as an energy reservoir, a layer of thermal insulation, tissue providing mechanical protection, and an endocrine organ [3,56]. Brown adipose tissue acts as an energy combustion site to maintain body temperature [3,56].

In adult humans, white adipose tissue represents more than 90% of fat and is distributed in the abdominal cavity around organs and the subcutaneous area [3,56]. White adipocytes each contain a single large lipid droplet and few mitochondria [3,56]. White adipose tissue stores excess energy in the form of triglycerides and makes up approximately 15% to 25% of total body weight in lean men and 30% to 40% of total body weight in lean women [56,57]. Under physiological conditions, white adipose tissue has anti-inflammatory effects and improves free fatty acid metabolism. However, in a state of obesity, white adipose tissue is a source of pro-inflammatory cytokines and causes oxidative stress, leading to endothelial dysfunction [56,57]. 

Brown adipose tissue represents only 1–2% of fat in adult humans and is mainly located in cervical, supraclavicular, axillary, paraspinal, mediastinal, and abdominal depots [3,58]. Brown adipocytes contain multilocular lipid droplets and a large number of cristae-dense mitochondria [3,58]. Uncoupling protein 1 (UCP1) is a key factor in non-shivering thermogenesis in brown adipocytes and is involved in the production of heat and dispersion of energy [3,58]. Several investigators have demonstrated that the amount and activity of brown adipose tissue are inversely associated with BMI and cardiovascular risk factors, suggesting anti-obesity and anti-diabetes properties of brown adipose tissue [59,60]. Indeed, Raiko J et al. showed that FMD was positively correlated with brown adipose tissue activity in subjects with normal weight [61]. In humans, brown adipose tissue mass and activity decrease with aging [62]. Therefore, the activation of brown adipose tissue has been investigated as a potential therapeutic target for obesity. An experimental study showed that β3-adrenergic receptor agonists improve endothelial function [63]. Recently, O’Mara AE et al. showed that a 4-week treatment with the β3-adrenergic receptor agonist mirabegron increased brown adipose tissue metabolic activity and brown adipose tissue volume and energy expenditure and led to significant improvements in insulin sensitivity, plasma high-density lipoprotein, and apolipoprotein A1 in healthy women [64]. However, there is no information on the effects of β3-adrenergic receptor agonists on endothelial function in humans. Further studies are needed to determine the effects of β3-adrenergic receptor agonists on endothelial function and the long-term effects of mirabegron on outcomes. 

Another subset of UCP1-positive cells, namely, beige adipocytes, are found in white adipose tissue [3,58]. White adipocytes can differentiate into brown-like adipocytes under the condition of cold stimulation or activation of sympathetic adrenergic receptors on adipocytes [3,58]. This phenomenon is known as browning. Beige adipocytes are present predominantly in subcutaneous fat depots and are occasionally detected in visceral depots [59]. Beige adipocytes have characteristics similar to those of brown adipocytes (multilocular lipid droplets, large number of mitochondria, high metabolic activity) and contribute to body temperature maintenance [3,58]. Experimental and clinical studies have shown that beige adipocytes have anti-obesity and anti-diabetes effects [65,66,67,68]. Browning of white adipose tissue has attracted much attention as a therapeutic target for obesity and related metabolic complications. Although recent research has significantly increased the understanding of the browning of white adipose tissue in mice, knowledge of the browning of white adipose tissue in humans is limited. There are significant differences in the beige adipocyte physiology between humans and rodents [69]. The differences between brown adipocyte physiology and beige adipocyte physiology and the potential of beige adipocyte differentiation in humans are still poorly understood [70]. Browning is a reversible phenomenon. Beige adipocytes lose UCP1 expression and mitochondrial density without persistent stimulation [71]. The expression of beige genes in subcutaneous fat is dysregulated in obese individuals [72,73,74]. In addition, there are methodological limitations in evaluating the activity of beige adipocytes in humans [56]. F-18 fluorodeoxyglucose positron-emission tomography-CT (18F-FDG-PET/CT) is a standard technique for detecting beige adipocytes [75]. However, the resolution of 18F-FDG-PET/CT is insufficient to distinguish beige adipocytes from brown adipose tissue. The development of a non-invasive and high-resolution imaging tool for evaluating the activity of beige adipocytes and expanding our knowledge of browning in humans is needed to establish the benefits of browning as a therapeutic option against obesity.

## 6. Putative Mechanisms of Obesity-Induced Endothelial Dysfunction

**Figure 4:**
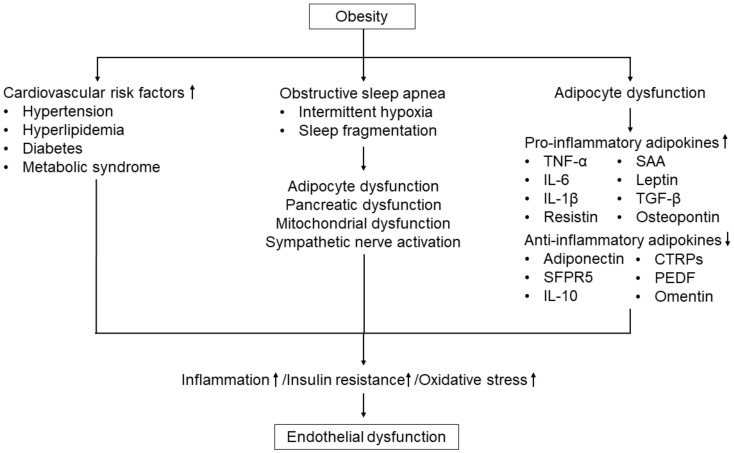
The putative mechanisms underlying endothelial dysfunction induced by obesity.

Obesity is one of the leading causes of endothelial dysfunction. Some possible mechanisms of obesity-induced endothelial dysfunction have been considered. The putative mechanisms of obesity-induced endothelial dysfunction are displayed in Figure 4.

Obesity is associated with the prevalence and development of hypertension, hyperlipidemia, diabetes, and metabolic syndrome [4,5,76,77,78,79,80]. These cardiovascular risk factors promote the production of ROS and induce endothelial dysfunction, as reviewed previously [7,81,82].

It is well known that obesity is a risk factor for the development of obstructive sleep apnea syndrome (OSAS), which is characterized by repeated episodes of obstruction of the respiratory passages during sleep that results in intermittent hypoxia and sleep fragmentation [83]. Intermittent hypoxia causes tissue hypoxia and promotes adipocyte dysfunction, pancreatic dysfunction, mitochondrial dysfunction, and overactivation of the sympathetic nervous system [84,85]. These comorbidities cause inflammation, insulin resistance, and oxidative stress and impair endothelial function. Several clinical studies have shown that endothelial function is impaired in patients with OSAS [86,87,88]. In addition, it has been reported that treatment with continuous positive airway pressure in OSAS patients is effective in improving endothelial function [89,90].

Obesity reflects increased adipose tissue. The amount of white adipose tissue is increased by 40% to 70% of total body weight in obese subjects [57]. Excess adipose tissue accumulation induces hypertrophy of adipocytes, and the formation of ectopic fat in the vasculature, liver, muscle, and heart [91]. Hypertrophy of adipocytes is associated with hypoxia in adipose tissue, leading to adipocyte dysfunction. Adipose tissue is a major source of pro-inflammatory and anti-inflammatory factors [3,48,92] In obesity, excess adipose tissue causes an increased production of pro-inflammatory factors such as tumor necrosis factor (TNF-α), interleukin (IL)-6, IL-1β, and resistin (Figure 2) [48]. These increased pro-inflammatory adipokines contribute to the development of inflammation, insulin resistance, and endothelial dysfunction [48,93,94].

Beneficial adipokines, particularly adiponectin, have anti-atherogenic, anti-diabetic, anti-inflammatory, and insulin-sensitizing functions [48,95,96]. Experimental studies have shown that adiponectin has protective effects on endothelial function through stimulation of adenosine monophosphate-activated protein kinase-dependent NO production [97,98]. Adiponectin increases cyclooxygenase-2 expression in endothelial cells, resulting in the enhancement of endothelial cell differentiation, migration, and survival [48]. Adiponectin has anti-inflammatory effects through inhibition of TNF-α-mediated nuclear factor kappa B (NF-κB) activation in endothelial cells and production of the anti-inflammatory cytokines (IL-10 and IL-1) [48,99]. Adiponectin promotes the transformation of macrophages from pro-inflammatory M1 to anti-inflammatory M2 [94]. Therefore, adipocyte dysfunction induces endothelial dysfunction through a decrease in the expression of adiponectin. Indeed, several clinical studies have shown that circulating adiponectin levels are positively correlated with endothelial function [100,101,102]. The imbalance in adipokine secretion in obesity promotes insulin resistance and inflammation, leading to the development of endothelial dysfunction.

## 7. Effects of Lifestyle Intervention on Endothelial Function in Obesity

Obesity is a modifiable risk factor for the development of cardiovascular events. Considering the putative mechanisms underlying the association of obesity with endothelial dysfunction, a reduction in weight should be effective for improving endothelial function. There have been numerous studies in which the association between lifestyle interventions for a reduction in weight and endothelial function was evaluated.

### 7.1. Diet

Some specific diets including the Mediterranean diet, ketogenic diet, and calorie restrictions have been shown to decrease body weight and decrease the incidence of cardiovascular events [103,104,105,106]. Recently, the Mediterranean diet has received considerable attention for its potential to prevent cardiovascular events [103,107]. It has been shown that the Mediterranean diet has beneficial effects on blood pressure, glucose, insulin resistance, lipid parameters, oxidative stress, and inflammation with a median weight loss of 1.72 kg {95% confidence interval (CI): −2.40 kg to −1.05 kg} [108]. Several investigators have shown that intervention with the Mediterranean diet improves endothelial function in healthy subjects [109], patients with cardiovascular risk factors [110,111,112,113], and patients with cardiovascular disease [114]. Yubero-Serrano EM et al. showed that a 1-year intervention with the Mediterranean diet decreased ROS production, cellular apoptosis, and endothelial cell senescence and improved endothelial function in coronary heart disease patients [114]. A meta-analysis revealed that intervention with the Mediterranean diet significantly improves endothelial function in patients with increased risk of cardiovascular disease, and even in healthy subjects and that the duration of the Mediterranean diet intervention is positively correlated with endothelial function [115]. Some studies have demonstrated that higher adherence to the Mediterranean diet could improve endothelial function [116,117]. These findings suggest that a long duration of and high adherence to the Mediterranean diet are favorable for endothelial function.

### 7.2. Physical Activity

Physical inactivity is one of the causes of obesity and is often targeted for intervention [118,119]. Exercise has many favorable effects on cardiovascular risk factors. The favorable effects include decreases in body mass index, blood pressure, and abdominal obesity and improvement in insulin sensitivity, liver function, lipid parameters, and inflammation [119,120,121,122,123,124]. A recent meta-analysis has shown that exercise has positive effects on body weight (mean weight loss ranging from 1.5 kg to 3.5 kg) and body fat (mean fat loss ranging from 1.3 kg to 2.6 kg) compared with a non-exercise control group in adults with overweight and obesity [125]. Clinical and experimental findings strongly suggested that exercise exerts endothelial protective effects through enhancement of NO synthase activity and anti-inflammatory action [126,127,128]. There have been numerous clinical studies in which the associations between exercise and endothelial function in people overweight or obese were evaluated [129,130,131,132,133,134]. However, the effects of exercise vary depending on the intensity, duration, and type of exercise (e.g., aerobic exercise, resistance training, and high-intensity interval training). Further studies are needed to examine effective algorithms of exercise for improving endothelial function.

### 7.3. Smoking Habits

Smoking is one of the risk factors for endothelial dysfunction, and endothelial function is impaired with an increase in smoking pack years [135]. It is well known that smoking is associated with weight loss and that smoking cessation leads to body weight gain. Nevertheless, smoking cessation is associated with a lower risk of cardiovascular events regardless of the degree of weight gain [136,137]. Several investigators have shown that smokers have abdominal obesity in comparison to non-smokers even though smokers have significantly lower BMIs [138,139]. Fukumoto K et al. showed that endothelial function is significantly improved after smoking cessation despite an increase in BMI. They also reported that an increase in FMD was not different among three therapy groups (varenicline, nicotine patch, and no pharmacotherapy groups) [140]. These findings suggested that smoking cessation is a worthwhile therapeutic approach for the management of obesity-associated complications.

### 7.4. Degree of Weight Loss with Lifestyle Intervention and Endothelial Function

Although there have been numerous studies on the reduction in body weight for preventing cardiovascular events, not all studies on weight loss have shown a reduction in cardiovascular events [141,142,143]. These results may be related to the limited differential weight loss between the intervention and control groups by the end of the trial. In addition, many individuals experience difficulty in maintaining weight reduction in the long term. A meta-analysis showed that weight loss increased FMD compared with that in controls by 3.29% (95% CI: 0.98–5.59%; *p* = 0.005; mean weight loss: 8.6 kg) and that each 10-kg decrease in body weight increased FMD by 1.11% (95% CI: 0.47–1.76%; *p* = 0.001) [144]. A post hoc analysis of the Look AHEAD study showed that weight loss of more than 10% in the first year was associated with an approximately 20% reduced risk of cardiovascular events, while there was no beneficial effect on the risk of cardiovascular events in subjects with weight loss of less than 10% [145]. The treatment of obesity may have different effects on cardiovascular outcomes depending on the degree and maintenance of weight loss.

## 8. Effects of L-Arginine Supplementation on Endothelial Function in Obesity

Amino acids have been considered to have some physiological roles in endothelial function. L-arginine is an endogenous amino acid and acts as a substrate for NO production in endothelial cells [146,147]. Obesity reduces L-arginine production and L-arginine transport, leading to impaired NO production and endothelial dysfunction [148]. Arginase is an enzyme of the urea cycle and cleaves L-arginine to form urea and L-ornithine. Activation of arginase can cause endothelial dysfunction by reducing L-arginine and NO production. Experimental and clinical studies have demonstrated that the induction of arginase activity in obesity is associated with endothelial dysfunction [147,149,150,151]. L-arginine supplementation has attracted much attention as a possible therapeutic option for endothelial dysfunction, through amelioration of NO production, insulin sensitivity, oxidative stress, and inflammation [152]. Indeed, a meta-analysis has revealed that supplementation of L-arginine for 8 weeks or more significantly decreases body weight {weighted mean difference: −0.71 kg (95% CI: −1.12 kg to −0.30 kg)} and waist circumference {weighted mean difference −2.02 cm (95% CI: −3.43 cm to −0.61 cm)} [153]. Additionally, Shiraseb F et al. reported that L-arginine supplementation significantly decreases systolic blood pressure {weighted mean difference: −6.40 mmHg (95% CI: −8.76 mmHg to −4.05 mmHg)} [154]. However, previous clinical studies have demonstrated that L-arginine supplementation does not have favorable effects on NO production and endothelial function [152,155,156]. There have been conflicting results regarding the effects of L-arginine supplementation on lipid metabolism and carbohydrates [152]. Additionally, it has been suggested that L-arginine supplementation increases mortality in patients with acute myocardial infarction [157]. A further understanding of the molecular mechanisms by which L-arginine supplementation affects endothelial function is needed to establish the therapeutic role of L-arginine supplementation in obesity.

## 9. Treatment of Obesity for Improvement of Endothelial Function

Some individuals with obesity do not respond to lifestyle interventions. Pharmacological and/or surgical interventions can be useful for the treatment of obesity in those individuals.

Bariatric surgery is an effective intervention for weight reduction in individuals with obesity. The AHA/ACC/TOS guideline recommends bariatric surgery in adults with BMI ≥ 40 kg/m^2^ or BMI ≥ 35 kg/m^2^ with obesity-related comorbidities [23]. A meta-analysis of studies on long-term outcomes of bariatric surgery has shown that weight loss reaches a peak at 2-year follow-up and is relatively stable from 2 to 20 years with a mean weight loss for this period of 24.8 kg [158]. Several investigators have shown that bariatric surgery significantly reduces cardiovascular risk factors and the incidence of cardiovascular events [159,160]. Bariatric surgery has been shown to improve endothelial function in subjects with obesity [161,162,163]. A meta-analysis has shown that percentage of changes in BMI is negatively correlated with endothelial function [163]. However, the short-term effects of bariatric surgery were evaluated in those studies and those studies were not randomized controlled studies. Indeed, Dantas WS et al. suggested that improvement in endothelial function is not sustained at 9 months after bariatric surgery [162]. Future long-term, randomized studies are needed to evaluate the effects of bariatric surgery, compared with the effects of optimal medical treatment, on endothelial function.

For individuals with BMI ≥30 kg/m^2^ or BMI ≥27 kg/m^2^ with obesity-related comorbidities, consideration should be given to pharmacotherapy along with lifestyle modification as a therapeutic option [23]. There are five Food and Drug Administration (FDA)-approved drugs for the treatment of obesity: pancreatic lipase inhibitor (orlistat), a combination of phentermine-topiramate (phentermine-topiramate), a combination of bupropion-naltrexone (bupropion-naltrexone), and glucagon-like peptide 1 (GLP-1) receptor agonists (semaglutide and liraglutide) [164,165]. A recent meta-analysis has shown that phentermine-topiramate {median weight loss of 8.07 kg (95% CI: −10.60 kg to −5.54 kg)} and GLP-1 receptor agonists {median weight loss of 4.96 kg (95% CI: −5.58 kg to −4.34 kg)} are the most effective drugs among anti-obesity medications for lowering weight [166]. When the weight loss effects of GLP-1 receptor agonists were examined separately, semaglutide had a considerable weight loss effect {median weight loss of 10.78 kg (95% CI: −12.38 kg to −9.19 kg)}, whereas liraglutide was less effective for weight loss {median weight loss of 4.51 kg (95% CI: −5.19 kg to −3.82 kg)} [166]. Once-weekly subcutaneous semaglutide has been shown to significantly decrease body weight in overweight or obese subjects with or without type 2 diabetes and it was approved by the FDA for treatment of obesity in June 2021 [167,168,169,170]. Experimental studies have shown favorable effects of GLP-1 on endothelial function through increasing endothelial NO phosphorylation and NO production and reducing the generation of ROS and inflammation [171,172]. In addition, a GLP-1 receptor agonist was shown to induce brown adipocyte differentiation in skeletal muscle in obese diabetic mice [173]. This finding suggests that a GLP-1 receptor agonist has beneficial effects on endothelial function by promoting brown adipocyte differentiation. However, the results of clinical studies regarding the effects of GLP-1 receptor agonists on endothelial function have been conflicting [174,175,176,177]. In addition, there is no information on the effects of semaglutide on endothelial function. In randomized clinical trials conducted in patients with type 2 diabetes, semaglutide showed superiority in body weight reduction compared to other glucose-lowering drugs [178,179,180]. Furthermore, semaglutide has been demonstrated to have cardiovascular and renal benefits in patients with type 2 diabetes [181,182,183]. Further investigations are needed to evaluate the relationship between semaglutide and endothelial function.

Despite the proven effectiveness of bariatric surgery and anti-obesity drugs for reducing weight, only 0.4% of eligible patients undergo bariatric surgery and the rate of prescriptions of anti-obesity medications remains very low (0.28% in 2016) [184,185]. In addition, a real-world study showed that adherence and persistence with anti-obesity drugs are poor [186]. These current data may show a missed opportunity for reduction in cardiovascular risk through comprehensive weight reduction strategies in obese individuals.

## 10. Future Challenges and Perspectives

Various pathways involved in obesity-induced endothelial dysfunction were described in this review. However, mechanisms underlying obesity-induced endothelial dysfunction remain incompletely understood. The development of molecular markers linked to obesity conditions and endothelial function is needed to provide new perspectives on the underlying mechanisms of obesity-induced endothelial dysfunction.

## 11. Conclusions

There is considerable evidence that obesity is associated with endothelial dysfunction through several mechanisms including oxidative stress, insulin resistance, and inflammation. The incidence of obesity-related cardiovascular diseases has been increasing worldwide. Additional focus should be given to the management of patients with obesity in order to reduce cardiovascular events.

## Data Availability

Not applicable.

## Abbreviations

ABSI, a body shape index; BMI, body mass index; CI, confidence interval; CT, computed tomography; CTRPs, C1q/TNF-related proteins; eNOS, endothelial nitric oxide synthase; FDA, Food and Drug Administration; FMD, flow-mediated vasodilation; GLP-1, glucagon-like peptide 1; NF-κB, nuclear factor kappa B; NO, nitric oxide; IL, interleukin; OR, Odds ratio; OSAS, obstructive sleep apnea syndrome; PEDF, pigment epithelium-derived factor; ROS, reactive oxygen species; SAA, serum amyloid A; SFPR, secreted frizzled-related protein; TGF, transforming growth factor; TNF, tumor necrosis factor; UCP1, uncoupling protein 1; and 18F-FDG-PET/CT, F-18 fluorodeoxyglucose positron-emission tomography-computed tomography.
